# Aesthetic evaluation underpinning brand love relationship development: an activation likelihood estimation meta-analysis and multivariate analysis

**DOI:** 10.3389/fnins.2024.1443578

**Published:** 2025-01-03

**Authors:** Shinya Watanuki

**Affiliations:** Department of Marketing, Faculty of Commerce, University of Marketing and Distribution Sciences, Kobe, Hyogo, Japan

**Keywords:** brand equity, default mode network, DMN, consumer neuroscience, neuromarketing, ALE

## Abstract

**Objectives:**

Brand love is a crucial construct in marketing strategies. Building brand love can generate stable profits for enterprises. Although the marketing literature points out that aesthetic factors contribute to establishing the relationship as a trigger, to what stage of the relationship do they influence the minds of consumers? The present study attempts to reveal the involvement of aesthetic experiences in brand love developmental dynamics.

**Methodology:**

Using the activation likelihood estimation method, we address this issue by assessing overlapping brain regions between brand love at each stage and aesthetic experiences. We adopted three major meta-analytic decoding analysis modules to objectively interpret these brain regions, namely, Neurosynth, NeuroQuery, and the Behavioral Analysis plugin (BrainMap platform). Moreover, we performed a correspondence analysis to identify relationships of mental processes between aesthetic experiences and brand love in each developmental stage of brand love.

**Findings:**

Our results suggest that the same neural mechanism and mental processes may be underlaid between brand love and aesthetic experiences across all stages. Although reward- and emotion-related mental processes are commonly underlaid between brand love at the first-half stage and aesthetic experiences, exteroceptive and interoceptive signals may drive those mental processes between the early and migration stages of brand love, respectively, and aesthetic experiences. Overlapping regions of brand love at the stable stage and aesthetic experiences may be associated with semantic processing.

**Conclusion:**

We demonstrate that several brain regions overlapped between brand love and aesthetic experiences across all the brand love developmental stages. Therefore, aesthetic experiences might be associated with the mental processes of brand love development through all the developmental stages. Our results suggest that aesthetic experiences are essential elements for developing brand-love relationships.

**Implications:**

Our findings indicate that marketers should recognize that aesthetic experiences play a crucial role in building a bond between brands and consumers, not only when choosing brands. Thus, marketers need to design visual strategies from the view of nurturing brand-love relationships.

## 1 Introduction

Brand equity is the most crucial asset for profitable success in the business market. Forming strong emotionally bonded relationships between brands and consumers leads to building valuable brand equity (Aaker, 1991; Keller, 1993). In even the digital age, brand equity plays a pivotal role in enhancing purchase intention and customer satisfaction (Ahmed et al., 2017a,2023a). Scholars refer to this relationship as brand love, which plays a key role (Batra et al., 2012). Much of the marketing literature reports that aesthetic attributes contribute to the formation of brand love, including loved objects (Ahuvia, 2005; Franzak et al., 2014; Warren et al., 2019; Bagozzi and Khoshnevis, 2022). Aesthetic appeals, such as a beautiful product design, play a role in triggering the generation of emotion and the emotional connection between brands and consumers (Candi et al., 2013). Pleasantness derived from the aesthetic attributes of products drives the buying motivation of consumers (Celhay and Trinquecoste, 2015). For example, Apple has been known as a brand with strong brand equity, because it has been ranked in the top tier of the ranking for brand value for several years (Kantar Millward Brown and WPP, 2024). Researchers point out that Apple products are designed with reference to the Braun product design (Mavroeidi, 2017; Zhang and Xue, 2019). Braun’s products were designed in collaboration with the Ulm School of Design, which has been inherited from the Bauhaus design philosophy (Mavroeidi, 2017; Zhang and Xue, 2019). Therefore, the product design employed by Apple can be considered an inheritance of the Bauhaus design. In fact, its aesthetic appeal influences the purchasing behavior of consumers (Warren et al., 2019). Notably, the aesthetic appeal of luxury brands functions as a social signal and reinforces the motivation for the self-expression of consumers (Bazi et al., 2020). According to Warren et al. (2019) and Bagozzi and Khoshnevis (2022), aesthetic appeal is one of the crucial components of brand coolness, which is the ascendant factor of brand love. Brand coolness contributes to forming a social signal in consumer minds. Social signals reduce risks in choosing brands under uncertain situations, reinforce relationships between brands and consumers, and eventually lead to enhancing brand equity (Ahmed et al., 2023b). Aesthetic experiences include these symbolic values (Markoviæ, 2012). Thus, aesthetically attractive brands have the possibility of enjoying these various benefits in brand choice behaviors.

In many cases, these aesthetic experiences seemingly play a role in the first stage of brand love in terms of design and appearance. However, brand love is dynamically developed and nurtured across several stages between brands and consumers, which is similar to romantic love relationships (Langner et al., 2016). In general, the dynamics of the development of brand love have four stages, namely, early, migration, stable, and decline (Langner et al., 2016). The early stage comprises the first stage of the relationship between brands and consumers, including chance encounters through advertisements on Facebook. Emotional factors at the migration stage facilitate and reinforce this relationship. Consumers regularly pick a particular brand without searching for other brands (stable stage), then eventually break up this relationship by gradually reducing purchases (decline stage). However, at which stage do aesthetic experiences involve brand love? If common mental processes would be underlaid between brand love and aesthetic experiences, what types of mental processes commonly function between both constructs at each stage?

Accordingly, although aesthetic experiences might contribute to developing the love relationships between brands and consumers, no studies have reported the dynamic involvement of aesthetic experiences in brand love. To address this research gap, the present study intends to elucidate the influence of aesthetic experiences on the mental processes related to the dynamics of brand love using a neuroimaging meta-analytic approach. Concretely, we identify common mental processes by revealing overlapping brain regions between brand love brain networks at each relationship stage and the core brain regions of aesthetic experiences.

## 2 Materials and methods

### 2.1 Neuroimaging meta-analysis approach

To infer the contributions of aesthetic experiences to brand love dynamics, we conducted a neuroimaging meta-analysis. We adopted an activation likelihood estimation (ALE) method (Eickhoff et al., 2009), which is one of the coordinate-based meta-analyses (CBMA) for the following reasons. First, CBMA can take activated peak coordinates from officially opened academic publications more easily than image-based meta-analyses (IBMA). Second, the ALE is the most prevalent method in the CBMA (Acar et al., 2018). Third, the results of the ALE were rigorously validated in comparison with the results of the IBMA (Salimi-Khorshidi et al., 2009).

### 2.2 Estimation technique

In the ALE method, the study calculated for the probabilities of collected peak coordinates by applying a three-dimensional Gaussian distribution function in each focus. To enhance the credibility of locations related to the activated peak coordinates, we tested these coordinates by comparing the probabilities of these coordinates with coordinates randomly generated by null distribution through appropriate permutation numbers. Eventually, we produced thresholded ALE maps on the basis of the peak coordinates that passed this test. Analyses were conducted using the following procedures. In the first step, we identified brain regions using the ALE. In the second step, we performed conjunction analysis to reveal the overlapping brain regions between brand love and aesthetic experiences. Finally, we performed decoding analysis to infer cognitive functions regarding the overlapping brain regions. The present study focuses on shared brain regions and mental processes between brand love dynamics and aesthetic experiences. Thus, we were unable to provide detailed assessments and considerations regarding the distinctiveness of each construct due to the limited word number.

### 2.3 Estimation procedure

#### 2.3.1 Brain regions related to brand love dynamics

We used the results of a previous study on brand love dynamics (Watanuki, 2022), which were calculated using the ALE method. The previous study classified brand love dynamics into four stages, namely, early, migration, stable, and decline. We collected publications based on the appropriate criteria according to the Preferred Reporting Items for Systematic Reviews and Meta-Analyses (PRISMA) guidelines and calculated the ALE maps related to each stage. However, the number of publications in the decline stage was extremely small for conducting ALE analysis. Eventually, we covered publications from the early to the stable stages of brand love. Using the ALE, we calculated the core brain regions related to brand love at each stage. We referred to this ALE analysis as the first ALE. After establishing the core regions as seed regions for reveal brain networks related to brand love at each stage, we executed the MACM (Robinson et al., 2010). We referred to this ALE as the second ALE. Please refer to Watanuki (2022) for the detailed conditions for collecting publications and parameters for ALE.

#### 2.3.2 Brain regions related to aesthetics

##### 2.3.2.1 Study collection method

**FIGURE 1:**
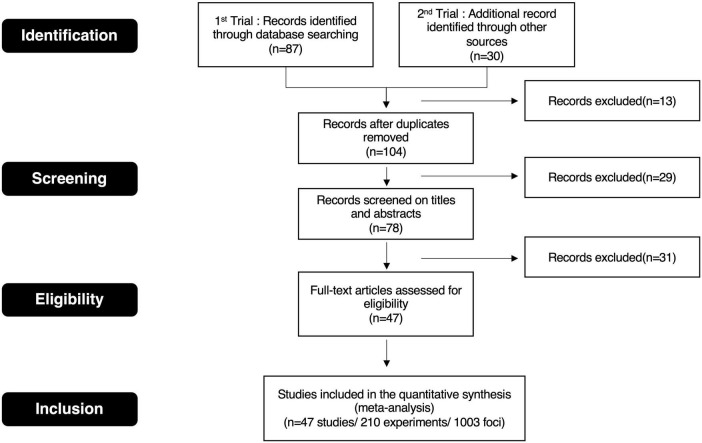
PRISMA flow diagram.

This neuroimaging meta-analysis was conducted to reveal the core brain regions of aesthetic experiences according to the PRISMA guidelines (Figure 1). We selected related studies from two databases, namely, PubMed^1^ and NeuroQuery (Dockès et al., 2020).^2^ We used different search words dependent on each database to collect various publications. In PubMed, we searched related publications using the following search words and conditions: “aesthetic” < AND > “neuroimage” < AND > “visual” < NOT > “eeg.” In NeuroQuery, we used “beauty” as the search word. The inclusion criteria were as follows: the articles were published in peer-reviewed journals written in English and published from January 2003 to January 2023. The studies were performed using positron emission tomography or magnetic resonance imaging. The activated coordinates were reported using the Montreal Neurological Institute (MNI) or Talairach reference space. Studies in which coordinates were not described were excluded. The participants were healthy. Positive valence stimuli were used in experiments; those using negative valence stimuli, such as sallow scenes and fearful faces, were excluded. Meta-analysis and case reports were excluded. The selected studies that met these criteria reached 47 (210 experiments, 1,003 foci).

##### 2.3.2.2 ALE method

Based on these foci, we conducted ALE using GingerALE 3.0.2.^3^ The parameters for ALE were set more conservatively than the recommended parameter criteria (Eickhoff et al., 2016) to reveal well-optimized brain regions engaged in aesthetic-related constructs (cluster-level FWE = 0.05, permutation number = 5,000, *p*-value = 0.001). The thresholded ALE maps were formatted with the NIfTI file format. We visualized the thresholded ALE maps using Mango.^4^ In the present study, foci reported by Talairach reference space were transformed into the MNI space using the icbm2tal (Lancaster et al., 2007) transform module provided by the BrainMap platform.

#### 2.3.3 Conjunction analysis

We conducted conjunction analysis using GingerALE 3.0.2^3^ to reveal the overlapping brain regions between brand love and aesthetic experiences. The parameters were set as follows: *p*-value = 0.01, permutation size = 10,000, minimum volume = 100 mm^3^.

### 2.4 Decoding analysis

#### 2.4.1 Decoding method

We decoded the overlapping brain regions between brand love and aesthetic experiences using the three representative decoding analysis modules [Neurosynth (Gorgolewski et al., 2015), NeuroQuery (Dockès et al., 2020), and Behavioral Analysis plugin (Lancaster et al., 2012)] to rigorously infer mental processes regarding overlapping brain regions. The Neurosynth cognitive decoding is one of the functions equipped with NeuroVault^5^ and covers 1,334 terms for decoding brain regions (Yarkoni et al., 2011). The region of interest (ROI) concerning the overlapping brain regions was uploaded into the NeuroVault and transferred to a cognitive decoding module. Afterward, the related terms with the ROI were produced. Posterior probability scores were calculated as the degree of matching between the ROI and terms. The NeuroQuery decoding model is provided with a Python program (Dockès et al., 2020) and prepares 5,144 terms for decoding brain regions. After the threshold ALE image was uploaded onto the NeuroQuery database, we produced terms matched the image using the NeuroQuery decoding model. Similarity scores were calculated as the degree of matching between inputted brain regions and terms. The Behavioral Analysis plugin is a decoding module provided by the BrainMap platform (Lancaster et al., 2012). It is provided in the Talairach brain space format; thus, we transformed the calculated ALE map formatted with the MNI brain space into the Talairach brain space format using the MNI-to-Tal transform function in Mango. Decoding analysis using the Behavioral Analysis plugin is performed according to the Behavioral Domains (i.e., Action, Cognition, Emotion, Interoception, and Perception) prepared by the BrainMap database. Each domain has subcategories for decoding: Action, Cognition, Emotion, Interoception, and Perception have 8 [“Execution (Speech),” “Execution (Unspecified),” “Imagination,” “Inhibition,” “Motor Learning,” “Observation,” “Preparation,” “Rest”], 17 [“Attention,” “Language (Orthography),” “Language (Phonology),” “Language (Semantics),” “Language (Speech),” “Language (Syntax),” “Language (Unspecified),” “Memory (Expliicit),” “Memory (Implicit),” “Memory (Working),” “Memory (Unspecified),” “Music,” “Reasoning,” “Social Cognition,” “Somatic,” “Spatial,” “Temporal”], 15 [“Intensity,” “Negative (Anger),” “Negative (Anxiety),” “Negative (Disgust),” “Negative (Embarrassment),” “Negative (Fear),” “Negative (Guilt),” “Negative (Punishment/Loss),” “Negative (Saddness),” “Negative (Unspecified),” “Positive (Humor),” “Positive (Happiness),” “Positive (Reward/Gain),” “Positive (Unspecified),” “Valence”], 11 (“Baroregulation,” “Gastrointestinal/Genitourinary,” “Heartbeat Detection,” “Hunger,” “Osmoregulation,” “Respiration Regulation,” “Sexuality,” “Sleep,” “Thermoregulation,” “Thirst,” “Vestibular”), and 9 [“Audition,” “Gustation,” “Olfaction,” “Somesthesis (Pain),” “Somesthesis (Unspecified),” “Vision (Color),” “Vision (Motion),” “Vision (Shape),” “Vision (Unspecified)”] categories, respectively. In contrast to the previous two decoding modules, the Behavioral Analysis plugin presents statistically significant decoded terms toward brain regions. Decoded terms with z-scores ≥ 3.0 are considered significant.

#### 2.4.2 Organization of the decoded terms into six metal processes

The Behavioral Analysis plugin defines decoded subcategories as “domain,” which pertains to comprehensive constructs. However, the decoded terms in Neurosynth and NeuroQuery are not organized into appropriate higher mental processes constructs. Although these terms are helpful for understanding specified constructs, inferring comprehensive mental processes may be challenging with the raw usage of these terms. Moreover, the expressed meanings of these constructs can be hardly interpreted due to the simple results produced by the machine learning techniques. To address this issue, the current study used the functions provided in Neurosynth and NeuroQuery. The decoded terms in Neurosynth and NeuroQuery are linked to related studies, we investigated the constructs related to these terms. These decoded platforms prepare functions for the easy interpretation of terms in each platform. Neurosynth prepares a topic-based meta-analysis module, which organizes terms for decoding into several topics as calculated using Latent Dirichlet Allocation (LDA) and a term-based meta-analysis module. Regarding the former approach, the eight topic groups are stored in the module as v4 topic 50, v4 topic 100, v4 topic 200, v4 topic 400, v5 topic 50, v5 topic 100, v5 topic 200, and v5 topic 400. For example, v4 indicates that the results were analyzed on the basis of the database for 2015, and v5 gives results based on the database as of 2018. A topic number, such as “50” and “200,” denotes the number of clusters automatically separated by LDA. A low topic number indicates that the terms are divided into rough inter-correlative term groups. We intend to infer a comprehensive construct concerning decoded terms; thus, we adopt v5 topic 50, which is the latest version. For example, we can interpret the decoded term “value” in Neurosynth by investigating to which clusters they belong, which belongs to topics 030 and 049. Topic 030 is composed of reward and decision-making related terms such as “decision,” “choice,” and “reward,” and topic 049 is composed of terms such as “depression,” “mdd,” and “mood.” Moreover, regarding the latter approach, “value” is associated with 470 studies. Each study displays loading values that express the degrees of a relationship between terms and studies. Regarding “value,” the study with the highest loading value was “The importance of actions and the worth of an object: dissociable neural systems representing core value and economic value (loading value = 0.656).” The study with the second highest loading value was “The decision value computations in the vmPFC and striatum use a relative value code that is guided by visual attention (loading value = 0.541).” These studies could be interpreted as a reward- and subjective value decision-making-related studies by assessing their abstracts and entire manuscripts. We executed this operation for the top 25 studies for each decoded term. Using these procedures, we eventually defined “value” as an RD-related construct. NeuroQuery also produces links to studies related to the decoded terms. Specifically, the cross-relational terms of the decoded terms are produced in the “In expansion” section of the NeuroQuery platform. For example, “default” produces “default mode” and “dmn” as cross-relational terms. Therefore, the decoded term “default” could be interpreted as belonging to a DMN-related mental process. Moreover, related studies are also produced in the “Publications related to the query” section of the platform. We inferred the mental processes related to the decoded terms using the same approach executed in Neurosynth, even if the cross-relational terms produced in the “In expansion” section are vague. Regarding these decoding platforms, we provide in-depth explanations in Supplementary Figures 2, 3. By performing these procedures for the decoded terms, we classified them into six mental process constructs.

#### 2.4.3 Correspondence analysis

**FIGURE 2:**
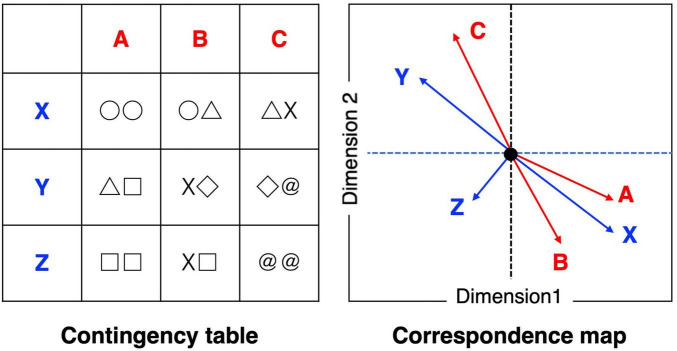
Simple conceptual explanations of correspondence analysis.

We conducted correspondence analysis to rigorously organize the decoded results for each decoding method using the R package “ca” (Nenadic and Greenacre, 2007). We can objectively infer the characteristic mental processes with which brain regions overlap between brand love in each stage and aesthetic experiences by analyzing the results of correspondence analysis. This approach is intended to reduce high-dimensional variables into low-dimensional ones using the singular-value decomposition technique. Notably, correspondence analysis is typically applied for a two-way contingency table data format. In this process, row and column coordinates are calculated, and these coordinates are simultaneously plotted on two-dimensional orthogonal axes. The results are interpreted based on the closeness between vectors, which are lined from the origin to each coordinate. We concisely describe conceptual explanations in Figure 2, concerning how to translate the results of correspondence analysis. The column (A, B and C) and row variables (X, Y, and Z) are reduced into two dimensions and the values, which represents characteristics of each dimension, are provided to these variables by calculating the contingency table. After calculating this contingency table, suppose that the correspondence map is produced. The way of interpreting the map is as follows. The A, B, and X variables are similar constructs. The C and Y are similar constructs. Accordingly, column variables A and B and row variables Y are plotted on opposite positions; therefore, the A, B, and Y variables can be presumed as bipolar constructs. The Z can be expressed as the relatively independent concept among variables.

## 3 Results

### 3.1 Activation likelihood estimation

#### 3.1.1 Brain regions related to brand love

The study input 4,799 foci, 330 experiments, and 5,780 subjects for the meta-analytic connectivity modeling (MACM) to reveal the brain networks related to brand love at the early stage. The basal ganglia (caudate head and body), limbic system (the parahippocampal gyrus and amygdala), anterior and mid insula, midbrain, and cortical midline structure [ventral medial prefrontal cortex (VMPFC), dorsal medial prefrontal cortex (DMPFC), and posterior cingulate cortex] were activated in the early stage. Brain regions composed of the reward network, the ventral insula pathway, and the default mode network (DMN) were also activated. For the migration stage, we input 2,296 foci, 157 experiments, and 2,934 subjects for the MACM. We observed the activation of the cortical midline structure, limbic system (the parahippocampal gyrus), anterior cingulate cortex (ventral and dorsal part), and dorsal part of the anterior insula and observed brain regions related to the DMN and salience network. In terms of the stable stage, we used 793 foci, 47 experiments, and 759 subjects and noted the activation of the dorsal striatum, anterior insula, inferior frontal gyrus (IFG), and parietal regions as well as brain regions related to several networks (the habit, salience, and dorsal frontal striatal networks, and the nigrostriatal dopamine pathway).

#### 3.1.2 Brain regions related to aesthetics

We conducted ALE analysis using a dataset composed of 1,003 foci, 210 experiments, and 3,941 subjects. Supplementary Table 1 presents the results. The activated brain areas were classified under nine cluster areas (Supplementary Table 2 and Supplementary Figure 1). Cluster 1 was composed of the occipital (inferior and middle occipital gyrus), posterior (lingual gyrus), temporal lobes (fusiform gyrus), and cerebellum (the culmen and declive). The medial frontal regions dominated clusters 2 and 3. Specifically, cluster 2 consisted of relatively ventral regions of the medial prefrontal cortex, while cluster 3 was activated in the dorsal parts of the medial prefrontal cortex. Clusters 4 and 8 were nearly dominated by the occipital lobe. The brain regions in cluster 5 correspond with Brodmann area (BA) 37 (BA37). BA37 consists of the parahippocampal and fusiform gyri. The IFG was the main brain region found in clusters 6 and 7. Although the claustrum was calculated as the peak-activated brain region in cluster 9, and BA13 dominated cluster 9. BA13 nearly corresponds to the insula. The current results are consistent with those of previous meta-analysis studies (Brown et al., 2011; Vartanian and Skov, 2014; Boccia et al., 2016; Chuan-Peng et al., 2020; Feng et al., 2021; Table 1) regarding the major activated brain regions such as the fusiform gyrus, VMPFC, DLPFC, and insula.

**TABLE 1:** Comparison between results of the current study and those of previous meta-analytical studies on brain regions related to aesthetic experiences.

Brain region at the reported peak coordinates	The present study	Brown et al., 2011	Vartanian and Skov, 2014	Boccia et al., 2016	Chuan-Peng et al., 2020	Feng et al., 2021
VMPFC (BA24/32)	X	X		X	X	X
MPFC (BA10)	X				X	X
DMPFC	X			X		
DLPFC (BA9/46)	X		X	X		X
Midcingulate cortex		X				
Posterior cingulate cortex			X			
Inferior frontal gyrus		X				
Middle frontal gyrus (BA6)		X		X		X
Superior frontal gyrus (BA10)						X
Precentral gyrus				X		X
inferior parietal lobule		X				
Precuneus (BA7)			X	X		
Inferior temporal gyrus			X			
Superior temporal gyrus			X			
Fusiform gyrus	X	X	X	X		X
Lingual gyrus	X		X			
Inferior occipital gyrus	X		X	X		
Middle occipital gyrus	X		X			
Claustrum/insula (BA13)	X	X	X	X		
Parahippocampal gyrus	X		X	X		
Amygdala		X		X		
Dorsal striatum		X	X			
Ventral striatum		X			X	
Thalamus		X				
Midbrain		X				
Cerebellum	X	X		X		

“X” indicates that activation was observed.

#### 3.1.3 Conjunction analysis: overlapping brain regions

**FIGURE 3:**
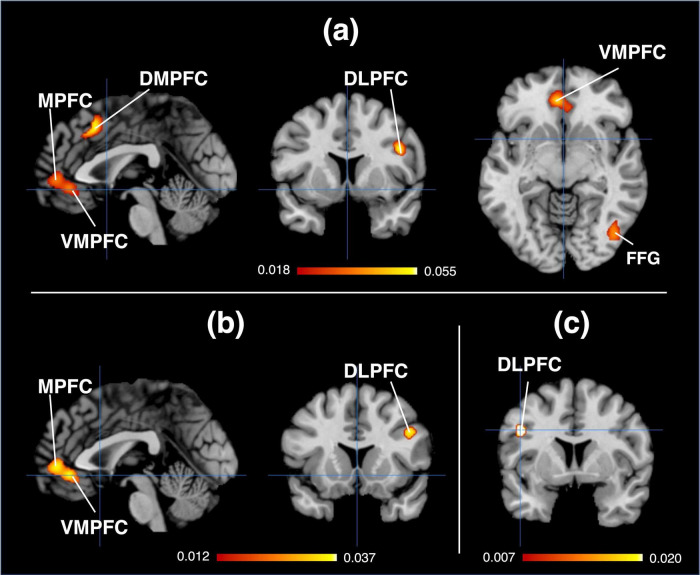
Overlapping brain regions. **(A)** Brand love in the early stage and aesthetic experiences, Crosshairs = (0.8–9). **(B)** Brand love in the migration stage and aesthetic experiences, Crosshairs = (0.14–8). **(C)** Brand love in the stable stage and aesthetic experiences, Crosshairs = (–46.6–30). DLPFC, dorsal lateral prefrontal cortex; DMPFC, dorsal medial prefrontal cortex; MPFC, medial prefrontal cortex; VMPFC, ventral medial prefrontal cortex; FFG, fusiform gyrus.

In the early stage of brand love and aesthetic experiences, the medial prefrontal regions from the ventral to dorsal parts were the major overlapping brain regions. The other overlapping brain regions were the fusiform gyrus, IFG (DLPFC and BA9), and the insula (BA13). In terms of brain regions at the migration stage and aesthetic experiences, the major overlapping brain regions were the relatively anterior and ventral parts of the medial prefrontal regions. The dorsal part of the prefrontal regions also overlapped. The dorsal lateral part of the prefrontal regions, which covered the IFG to the precentral gyrus, was observed as the overlapping brain regions in brand love at the stable stage and aesthetic experiences. Figure 3 and Table 2 present the results.

**TABLE 2:** Overlapping brain regions between brand love and aesthetic experiences.

Cluster	Side	Brain region	BA	Peak coordinates			ALE values	Cluster size (mm^3^)
				**x**	**y**	**z**		
**Overlapping brain regions: Early stage of brand love & aesthetic experiences**								
1	L	Anterior cingulate (VMPFC)	BA24	−4	40	−8	0.0492	4,520
	L	Medial frontal gyrus (MPFC)	BA10	−4	54	−2	0.0475	
	R	Anterior cingulate (VMPFC)	BA24	6	34	−10	0.0310	
2	L	Medial frontal gyrus (DMPFC)	BA6	2	18	46	0.0544	2,416
	L	Cingulate gyrus (DMPFC)	BA32	0	18	42	0.0540	
	L	Medial frontal gyrus (DMPFC)	BA8	−6	24	44	0.0443	
3	R	Fusiform gyrus	BA19	44	−72	−10	0.0398	2,184
	R	Fusiform gyrus	BA37	40	−52	−18	0.0369	
4	R	Inferior frontal gyrus (DLPFC)	BA9	46	12	26	0.0734	1,808
5	R	Claustrum	—	34	22	0	0.0374	1,248
**Overlapping brain regions: Migration stage of brand love & aesthetic experiences**								
1	L	Anterior cingulate (VMPFC)	BA24	−4	40	−8	0.0492	5,040
	L	Medial frontal gyrus (MPFC)	BA10	−4	56	−2	0.0461	
	R	Anterior cingulate (VMPFC)	BA24	6	34	−10	0.0310	
2	R	Inferior frontal gyrus (DLPFC)	BA9	46	18	26	0.0403	880
	R	Inferior frontal gyrus (DLPFC)	BA9	46	10	30	0.0346	
**Overlapping brain regions: Stable stage of brand love & aesthetic experiences**								
1	L	Precentral gyrus (DLPFC)	BA6	−46	4	30	0.0263	480

BA, Brodmann area; DLPFC, dorsal lateral prefrontal cortex; DMPFC, dorsal medial prefrontal cortex; MPFC, medial prefrontal cortex; VMPFC, ventral medial prefrontal cortex.

### 3.2 Decoding analysis regarding overlapping brain regions between brand love dynamics and aesthetic experiences

**FIGURE 4:**
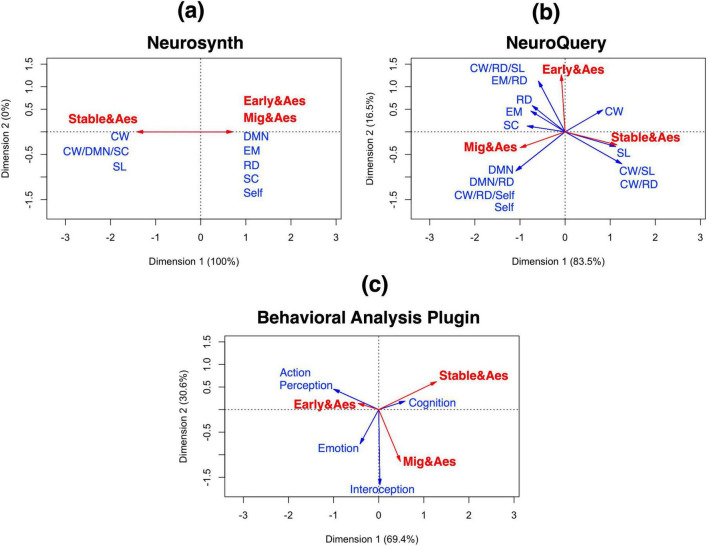
Correspondence map concerning the decoded results based on each decoding analysis module. **(a)** Neurosynth. **(b)** NeuroQuery. **(c)** Behavioral Analysis Plugin. Red vectors represent row vectors. Blue vectors represent column vectors. Early&Aes, overlapping brain regions between the early stage of brand love and aesthetic experiences; Mig&Aes, overlapping brain regions between the migration stage of brand love and aesthetic experiences; Stable&Aes, overlapping brain regions between the stable stage of brand love and aesthetic experiences; CW, cognitive control and working memory; DMN, default mode network; EM, emotion; RD, reward-based decision-making; SC, social cognition; Self, self-referential; SL, semantic and language.

Supplementary Table 3 lists the detailed information. We adopted the top fifteen ranked terms in the Neurosynth cognitive decoding module (Gorgolewski et al., 2015) and in the NeuroQuery decoding model (Dockès et al., 2020). Furthermore, we determined statistically significant terms (z-scores ≥ 3.0) in the Behavioral Analysis plugin according to Lancaster et al. (2012). We show these top five ranked terms and significant categories in Table 3. The decoded terms in the Neurosynth cognitive decoding and the NeuroQuery decoding model did not define these as appropriate comprehensive constructs. Thus, we organized them into six comprehensive mental processes according to the operations described in the “2. Materials and methods” section: cognitive control and working memory (CW), reward-based decision-making (RD), DMN, self-referential (Self), social cognition (SC), and semantic and language (SL)-related mental processes. Although the majority of the decoded terms were defined under these six mental processes, we did not classify several terms into identical mental processes. These terms were interpreted as constructs composed of multiple metal processes. For example, any term could be translated as a construct composed of CW- and RD-related mental processes; thus, we described it as CW + RD. Moreover, we defined the terms used in various and general contexts as “undefined (UD).” Regarding the Behavioral Analysis plugin, we did not dually and newly re-define decoded terms as mental process constructs, because it previously defined comprehensive mental process constructs as “domain.” Table 4 presents the total number of mental processes based on the results. Moreover, we performed correspondence analysis using data in Table 4, excluding the UD construct. Figure 4 depicts the correspondence map, which is a result of correspondence analysis.

**TABLE 3:** Decoded results at the top 5 terms and categories in each decoding analysis module.

Neurosynth				NeuroQuery				BAP (brain map)		
**R**	**Term**	**MP**	**Corr**	**R**	**Term**	**MP**	**Sim**	**Category**	**Domain**	**Z**
**Overlapping brain regions: Early stage of brand love & aesthetic experiences**										
1	Value	RD	0.237	1	Choice	RD	0.94	Attention	Cognition	14.216
2	Money	RD	0.217	2	Conflict	CW	0.82	Reasoning	Cognition	11.061
3	Midline	Self	0.190	3	Cognitive control	CW	0.8	Positive (Reward/Gain)	Emotion	10.295
4	Referential	Self	0.155	4	Borne	RD	0.69	Language (Semantics)	Cognition	10.066
5	Default	DMN	0.154	5	Evaluation	EM/RD/CW	0.67	Memory (Working)	Cognition	8.904
**Overlapping brain regions: Migration stage of brand love & aesthetic experiences**										
1	Value	RD	0.246	1	Choice	RD	0.75	Positive (Reward/Gain)	Emotion	6.409
2	Money	RD	0.207	2	Borne	RD	0.65	Reasoning	Cognition	6.338
3	Midline	Self	0.198	3	Trait	UD	0.6	Attention	Cognition	4.988
4	Referential	Self	0.154	4	Social cognitive	SC	0.55	Negative (Fear)	Emotion	4.012
5	Default	DMN	0.149	4	Default	DMN	0.55	Memory (Expliicit)	Cognition	3.715
**Overlapping brain regions: Stable stage of brand love & aesthetic experiences**										
1	Similarity	SM	0.361	1	Left		1	Memory (Working)	Cognition	4.868
2	Engagement	UD	0.243	2	Task	CW	0.98	Language (Semantics)	Cognition	4.406
3	Letter	SM	0.232	3	Syntactic	SM	0.81	Reasoning	Cognition	4.394
4	Color	UD	0.170	4	Demand	CW	0.79	Language (Phonology)	Cognition	4.291
5	Performance	CW	0.169	4	Switching	CW	0.79	Language (Speech)	Cognition	4.011

BAP, Behavioral Analysis plugin; R, rank; MP, mental process constructs; Corr, correlation coefficients; Sim, similarity score; Z, z-score; CW, cognitive control and working memory; DMN, default mode network; EM, emotion; RD, reward-based decision-making; SC, social cognition; Self, self-referential; SL, semantic and language; UD, undefined.

**TABLE 4:** Contingency table based on mental processes/domains in each decoding analysis module (excluding the undefined construct).

Mental processes	Early & aesthetic	Mig & aesthetic	Stable & aesthetic
**Neurosynth**			
CW	0	0	8
CW/SL	0	0	1
DMN	2	2	0
EM	2	2	0
RD	4	4	0
SC	1	1	0
Self	4	4	0
SL	0	0	4
**NeuroQuery**			
CW	5	0	11
CW/SL	0	0	7
CW/RD/SL	1	1	0
EM/RD	1	1	0
CW/RD	0	0	1
DMN	0	7	0
DMN/RD	0	3	0
EM	2	4	0
RD	4	7	0
SC	1	3	0
Self	0	3	0
CW/RD/Self	0	2	0
SL	1	0	9
**Behavioral Analysis plugin (brain map)**			
**Domain**	**Early & aesthetic**	**Mig & aesthetic**	**Stable & aesthetic**
Action	4	0	0
Cognition	11	4	7
Emotion	5	2	0
Interoception	1	1	0
Perception	6	0	0

Early & aesthetic, overlapping brain regions between the early stage of brand love and aesthetic experiences; Mig & aesthetic, overlapping brain regions between the migration stage of brand love and aesthetic experiences; Stable & aesthetic, overlapping brain regions between the stable stage of brand love and aesthetic experiences; CW, cognitive control and working memory; DMN, default mode network; EM, emotion; RD, reward-based decision-making; SC, social cognition; Self, self-referential; SL, semantic and language.

#### 3.2.1 Early stage of brand love and aesthetic experiences

Neurosynth decoded RD mental process-related terms (“value,” “money,” “smoking,” and “rewards”) in the top tier. Although “value” was included in two topic clusters, we defined it as an RD-related mental process by referring to the topic-based meta-analysis module and assessing related studies (the “2 Materials and methods” section provides the details). Although no topic clusters covered “money,” it was classified under RD by assessing related studies. “Smoking” belonged to topic 013, which is composed of addiction and reward modality-related terms such as “cocaine” and “cannabis,” we defined it as RD. Self-related terms (e.g., “midline,” “referential,” “self-referential,” and “subjective”) were ranked in the second tier, and DMN-related terms (“default” and “default mode”) were in the third tier. Two decoded terms (“engagement” and “personal”) were classified under UD, because defining these terms under the six constructs was challenging due to their usage under various contexts. No topic clusters included “engagement.” The term “personal” belonged to four topic clusters. Therefore, RD and self-related mental processes were dominantly decoded.

Regarding decoded results using NeuroQuery, two constructs were mainly decoded, namely, mental processes related to RD (e.g., “choice,” “borne,” “economic,” and “game”) and CW (e.g., “conflict,” “cognitive control,” “sorting,” “stroop,” and “Wisconsin”). As for the second tier, terms related to EM (“emotional responses” and “negative emotion”) were decoded. “Norm” could be classified under SL, because “semantic” was produced as a cross-relational term. Two decoded terms (“inappropriate,” and “evaluation”) were interpreted as constructs across multiple mental processes, thus, we could not classify them under one identical construct. The term “inappropriate” could be considered CW + SL + RD, and “evaluation” could be considered RD + EM. The term “inappropriate” produced four cross-relational terms (i.e., “motor,” “task,” “semantic,” and “reward”). The majority of the cross-relational terms for “motor” were brain region-related terms such as “m1,” and “sma”; therefore, it was not beneficial for defining an appropriate construct of “inappropriate.” However, cross-relational terms for “task” were associated with “working” and “working memory.” These terms could be interpreted as constructs related to working memory; as such, we eventually defined “inappropriate” as CW, SL, and RD. The term “evaluation” produced “pain” and “reward” as cross-relational terms. For this reason, we defined “evaluation” as RD and EM. Given that the other decoded terms (“mother,” “setting,” “trait,” “rule,” and “strategy”) were used in various contexts, we classified them under UD. Therefore, NeuroQuery dominantly decoded the mental processes related to CW and RD. Given the multiple constructs, the RD-related mental process was strongly decoded in this stage.

Regarding the decoded results using the Behavioral Analysis plugin, Cognition [“Attention,” “Reasoning,” and “Language (Semantics)”] and Emotion [“Positive (Reward/Gain)”] domains were placed at the top tier. All domains (Action, Cognition, Emotion, Interoception, and Perception) were significantly decoded. Notably, in contrast to the other stages, many categories under the Perception domain [“Vision (Shape/Unspecified),” “Somesthesis (Pain/Unspecified),” “Audition,” and “Gustation”] were decoded.

#### 3.2.2 Migration stage of brand love and aesthetic experiences

The terms decoded using Neurosynth were nearly similar to those regarding the early stage of brand love and aesthetic experiences. For mental processes, the early and migration stages produced entirely equal results (Table 4).

NeuroQuery decoded many distinctive terms that differed from those by Neurosynth, excluding several decoded terms. The following terms for five major mental processes were explicitly decoded as follows: RD (“choice,” “borne,” “price,” “gift,” “abuse,” “products,” and “willingness”), SC (“social cognitive” and “altruistic”), DMN (“default network,” “thought,” “network dmn,” “induction,” and “dmn”), Self (“self-referential,” “referential,” “midline,” “health behavior,” and “sedentary”), and EM (“emotional responses,” “mood,” and “positive negative”). We could not classify the following six terms under appropriate identical constructs. “Evaluation” was classified under RD + EM, and “inappropriate” was classified under CW + SL + RD. The term “impact” was considered a construct across two constructs (RD + DMN), because it produced cross-relational terms such as “reward,” “dmn,” and “default.” The terms “health behavior” and “sedentary” were interpreted as CW + Self + RD, because they produced “task” and “reward” as cross-relational terms, and many self-related studies were queried. Lastly, classifying the three other terms (i.e., “trait,” “mother,” and “public”) under any appropriate constructs was difficult, because these terms were used in various contexts. Thus, mental processes related to DMN and RD were dominantly decoded in this stage.

The Behavioral Analysis plugin decoded three domains, namely, Emotion [“Positive” (Reward/Gain)], Cognition [“Reasoning,” “Attention,” “Memory (Explicit),” and “Social Cognition”], and Interoception (“Sexuality”). In contrast to the decoded results of the previous stage, the Perception and Action domains were non-significant.

#### 3.2.3 Stable stage of brand love and aesthetic experiences

Neurosynth decoded many terms related to cognitive loading tasks such as “control task,” “demand,” “difficulty,” “interference,” “performance,” “stroop,” and “switch.” These terms may be associated with mental processes related to CW. For example, given that “stroop” is classified under topic 020, which includes terms related to cognitive control such as “control,” “conflict,” and “executive.” The term “stroop” is also associated with the Stroop task, which is used for assessing mental processes related to cognitive control. SL-related terms, such as “letter,” “phonological,” “similarity,” and “verbal” were also decoded. For example, “similarity” is classified under topic 038, which is composed of terms such as “semantic,” “knowledge,” and “representation”; thus, we defined it as SL. The term “judgment” was classified under Topic 008, which includes terms across several constructs such as “social,” “reasoning,” “mentalizing,” and “cognitive.” Thus, we defined it as CW + DMN + SC. The terms “color,” “engagement,” “forms,” and “personal” were classified under UD, because identifying these terms under any appropriate construct was challenging.

In NeuroQuery, mental processes related to CW and SL were mainly decoded. “Task” is associated with CW according to the results of cross-relational terms (e.g., “working,” “working memory,” and “memory”). We defined “accuracy,” “demand,” “distractor,” “dual,” “reaction time,” “resource,” “switching,” “task difficulty,” and “task switching” as CW, because “task” was the top cross-relational term. “Task” was correlated to CW-related terms (e.g., “working memory” and “working”). Although “letter” did not produce “task” in the list of cross-relational terms, it has many CW-related terms such as “working,” “working memory,” and “memory.” Thus, we defined it as CW. The terms “semantic,” “semantic processing,” and “language” could be evidently associated with the SL mental process, these terms were classified under SL. The terms “German,” “list,” “meaning,” “phonological,” “syntactic,” “word” and “syntax” produced cross-relational terms related to SL, such as “semantic” and “language.” Therefore, we defined them as SL. The terms “difficult,” “fluency,” “generation,” “judgment task,” “task demands,” “verbal fluency,” and “vocabulary” produced CW- and SL-related terms as cross-relational terms. For example, “generation” produced “language” and “task” as cross-relational terms and “vocabulary” led to “language” and “working memory.” Thus, these terms were classified as CW + SL.

Taken together, the decoding methods of Neurosynth and NeuroQuery mainly decoded overlapping brain regions as CW- and SL-related terms.

Regarding the decoded results using the Behavioral Analysis plugin, only categories in the Cognition domain [“Memory (Working),” “Language (Semantics),” “Reasoning,” “Language (Phonology),” “Language (Speech),” “Attention,” and “Language (Orthography)”] were decoded.

#### 3.2.4 Correspondence analysis: characteristics of mental processes in overlapping brain regions between brand love stages and aesthetic experiences

Figure 4 presents the results of correspondence analysis. In Neurosynth, only one dimension was calculated. All vectors were parallelly plotted on a vertical axis. The coordinates of overlapping brain regions at the first half stage and those of mental processes, such as DMN, EM, RD, SC, and Self, were plotted at the same plus value positions. In terms of coordinates of overlapping brain regions at the stable stage and those of mental processes, such as the CW, SL, and CW + DMN + SL, were plotted on the other side. These bipolar positions may express entirely opposite concepts. In other words, the same mental processes may be underlaid in the overlapping brain regions at the early and migration stages and aesthetic experiences. On the contrary, the overlapping brain regions of brand love at the stable stage and aesthetic experiences may be underlaid by distinctive mental processes that differ from those of brand love at the first half stage and aesthetic experiences. Therefore, the correspondence map in Neurosynth suggests that the overlapping brain regions of brand love at the first half stage and of aesthetic experiences may share anti-cognitive control processing with minimum or without cognitive load. However, cognitive control processing with cognitive load may underlie those of brand love at the stable stage and of aesthetic experiences.

Regarding the correspondence map in NeuroQuery, the vector of the early stage of brand love and aesthetic experiences was the closest to RD vectors. This finding suggests that the overlapping brain regions between the early stage of brand love and aesthetic experiences may be strongly associated with the RD mental process. The vector of the DMN and Self, including DMN + RD and CW + Self + RD, were the closest vector group toward the vector of the migration stage of brand love and aesthetic experiences. This result implies that DMN and Self-related mental processing may be commonly underlaid between brand love at the migration stage and aesthetic experiences. The vector of the stable stage of brand love and aesthetic experiences was the closest to the vector of the SL. The shared brain regions between brand love at the stable stage and aesthetic experiences may engage in SL-related mental processing. The vector of the first half stage of brand love and aesthetic experiences was closer to the vector of EM-related construct than the vector of the stable stage of brand love and aesthetic experiences. This notion indicates that the brain regions of the first half of brand love and aesthetic experiences may have a propensity for engaging in emotional processing instead of those of the stable stage of brand love and aesthetic experiences.

As for the correspondence map of the Behavioral Analysis plugin, the vector of the early stage of brand love and aesthetic experiences was the closest to the vector of the Action and Perception domains. Moreover, the vector of brand love at the migration stage and aesthetic experiences was the closest to the vector of the Interoception domain. At the stable stage, the vector of brand love and aesthetic experiences was the closest to the vector of the Cognition domain. These results indicate that each overlapping brain region may be associated with each mental process expressed by each vector of a domain. Moreover, the EM vector was plotted between the vectors of the early and migration stages of brand love, thus, their brain regions may exhibit a tendency to engage in emotion processing, which is in contrast to those of the stable stage of brand love and aesthetic experiences.

## 4 Discussion

To the best of our knowledge, this study is the first study to reveal the involvement of aesthetic experiences with brand love dynamics. Many brain regions overlapped between aesthetic experiences and brand love, although they were characteristic regions depending on each brand love developmental stage.

The anterior part of the VMPFC (aVMPFC) and IFG (DLPFC and BA9) were commonly activated in the overlapping brain regions between brand love and aesthetic experiences in the first half (early and migration stages). The aVMPFC has been known as the center of computing value information by integrating reward and emotion processing (Sescousse et al., 2013; Clithero and Rangel, 2014). Additionally, the aVMPFC is the core region of the DMN (Damoiseaux et al., 2008; Andrews-Hanna, 2012), which is associated with self-referential processing, mind wandering, and SC (Damoiseaux et al., 2008; Andrews-Hanna, 2012). Many brain regions overlap between the DMN and hedonic system, including reward and emotion processing (Acikalin et al., 2017). The DLPFC functions when making decisions and planning as one of the parts of the CW network and plays a crucial role in operating self-related inward-generated information in cooperation with the DMN brain regions (Northoff et al., 2006). These interpretations are consistent with the decoded results. Regarding the overlapping brain regions between brand love and aesthetic experiences during the first half stage, mental processes related to reward, emotion, self-referential, and DMN were characteristically decoded using the Neurosynth and NeuroQuery decoding methods. The Behavioral Analysis plugin also decoded reward and emotion-related constructs. Thus, although the reward aspects in the early stage of brand love and aesthetic experiences are slightly stronger than those in the migration stage, mental processes, which were integratively woven by the hedonic, subjective, and decision-making systems, underlaid those of the first half.

However, activation in overlapping brain areas between the early stage of brand love and aesthetic experiences was the broadest among the three stages. In particular, the brain regions of the ventral visual pathway [associated with bottom-up visual sensory processing (Kravitz et al., 2013)] and the DMPFC were activated during the early stage. The connection among the DMPFC, insula, and IFG engages in the integration of various sensory modalities and the generation of familiarity (Taylor et al., 2009a,2009b). The Behavioral Analysis plugin distinctively decoded somatosensory processing-related constructs in the overlapping brain regions at the early stage. Therefore, aesthetic appeal with pleasurable sensory impacts contributes to building love relationships between consumers and brands during the early stage (Warren et al., 2019). Alternatively, although activations in the aVMPFC and IFG were observed at the migration stage, the ventral pathway and DMPFC were not activated. These different activated regions seemingly contribute to different decoded results. NeuroQuery distinctively decoded DMN- and Self-related terms at the migration stage, although no somatosensory processing-related constructs were decoded. The different decoded results in the early and migration stages suggest that exteroceptive feelings play a crucial role in the mental process at the early stage of brand love and those of aesthetic experiences, but inward mental processes may dominate those of brand love at the migration stage and aesthetic experiences. The decoded results of the Behavioral Analysis plugin at the migration stage indicate that the mental processes of the migration stage of brand love and aesthetic experiences may be associated with interoceptive processing. Interoceptive signals generate a sense of self (Quigley et al., 2021). Similar to the medial side of the prefrontal regions, the DLPFC is associated with an inward-focused self-referential mental process. The DLPFC cooperates with brain regions of the DMN, while self-referential thoughts, such as autobiographical planning (Spreng et al., 2010) and goal-directed mental simulation, that internal resources are required (Gerlach et al., 2011). Schmid and Huber (2019) demonstrated that self-related constructs enhance love relationships between brands and consumers at the migration stage. The congruence between self and brand concepts is crucial in driving these relationships (Escalas and Bettman, 2015). These mental processes of consumers are spontaneously generated without external stimuli such as marketing initiatives (Batra et al., 2012). Regarding aesthetic experiences, the DMN is associated with a stimulus-independent deep moving for highly rated beautiful artworks (Vessel et al., 2012; Cela-Conde et al., 2013; Belfi et al., 2019). The activation of the DMN regions was observed for self-relevant aesthetic experiences such as self-imagination in viewing artworks (Kreplin and Fairclough, 2015). In this manner, self-referential and internally attentional thoughts may underlie the mental processes of brand love at the migration stage and aesthetic experiences.

Based on these considerations, although the common brain regions of the first half stage of brand love and aesthetic experiences may be involved in emotion- and reward-related mental processes, outward cues may drive the mental processes of the early stage of brand love and aesthetic experiences. Conversely, inward signals may enhance the mental processes of the migration stage of brand love and aesthetic experiences.

Regarding the overlapping brain regions of brand love at the stable stage and those of aesthetic experiences, the study observed the activation of the DLPFC. The activated overlapping brain regions among the three stages were the smallest areas. The results indicate that the three decoding analysis modules decoded terms related to CW and SM; thus, this region is associated with top-down processing (Menon, 2015) and integrates self-knowledge, which is distributed across brain areas when cognitive resource operation, such as decision-making and recall, is required (Northoff et al., 2006). The activation of the DLPFC was observed in aesthetic judgment, that is, a task for determining whether abstract graphics are beautiful or symmetric (Jacobsen et al., 2006). The brain regions related to the DLPFC and semantic knowledge, such as the parietal and temporal regions, were co-activated when the subjects imagined certain aspects of famous brand logos with sufficient self-knowledge (Chen et al., 2015). Their results were considered in accordance with parallel distributed processing theory (McClelland et al., 1987). McClure et al. (2004) demonstrated that the DLPFC was activated when using a brand logo with rich self-knowledge but not when using it in the opposite case. Typography and symbols constitute the majority of brand logos; thus, they can also be considered one type of abstract graphic. Given that brand logos are used as an experimental stimulus in many cases of consumer neuroscience, the overlapping brain regions between brand love at the stable stage and aesthetic experiences may be involved in semantic processing related to these abstract graphics. Thus, the DLPFC may play an essential role in integrating the brand’s distributed semantic memories and the symbolic meanings based on subjective aesthetic experiences by functioning as brand logos as cues. Aesthetic attractiveness is one factor that affects the perceived value of the brand (Shi et al., 2021). Because perceived values directly influence customer satisfaction (Ahmed et al., 2017b), enhancing the aesthetic attractiveness of brands causes customer satisfaction. According to Franzak et al. (2014), the symbolic meanings of aesthetic attractiveness engage in tightening engagements between brands and consumers, including customer satisfaction. Therefore, brand logos can be considered a trigger that activates brand-related stable and solid expressions composing aesthetically symbolic factors and self-knowledge. Integrating the aesthetic-derived symbolic factors and brand-related self-knowledge might be associated with building stable and long-lasting brand-love relationships between brands and consumers.

### 4.1 Limitations and future research

Although we assessed common mental processes between brand love and aesthetic experiences by assessing overlapping brain regions, the present study has its limitations. We were unable to assess variations in the activated brain regions according to reward modalities since we did not classify experiments for the current meta-analysis according to each reward modality. Several studies, including experimental and meta-analytical ones, demonstrate that various parts of the frontal brain regions are activated according to reward modalities such as primary and secondary rewards (Sescousse et al., 2010, 2013; Bartra et al., 2013; Clithero and Rangel, 2014). This finding is also confirmed in aesthetic contexts (Huang et al., 2016; Skov, 2019); thus, this issue should be addressed henceforth. Previous marketing studies report that the mental processes of consumers may be distinctive according to product categories in terms of detailed views, although their mental processes can be theoretically generalized (Howard and Sheth, 1969; Kollat et al., 1970). However, we did not select studies by each product category; thus, the present study could not assess variations in brain activation dependent on the product category. Regarding the overlapping brain regions between aesthetic experiences and brand love relationships at the stable stage, although our study interpreted that the integration of symbolic meanings with self-knowledge may lead to long-lasting brand love relationships, these cognitive functions may not be exclusive to the evaluation of abstract graphics, including brand logos, but be general mental processes related to semantic processing (Bara et al., 2022). This study could not address this issue. Moreover, we could not assess brain regions related to brand love at the decline stage due to insufficient studies. As such, further studies are required to complement these limitations.

## 5 Conclusion

**FIGURE 5:**
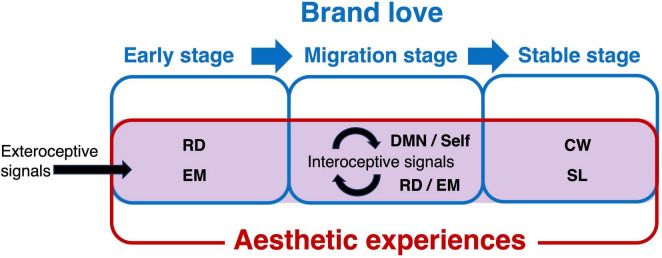
Common mental processes between brand love dynamics and aesthetic experiences. The blue rectangles represent brand love dynamics, the red rectangle represents aesthetic experiences, and the rectangle filled with light purple represents an area of common mental processes between brand love dynamics and aesthetic experiences. CW, cognitive control and working memory; DMN, default mode network; EM, emotion; RD, reward-based decision-making; SC, social cognition; Self, self-referential; SL, semantic and language.

In summary, the present study demonstrated that the neural mechanisms and mental processes of brand love dynamics and aesthetic experiences are shared, although the overlapping brain regions between them were gradually reduced under the proceeding love relationships between consumers and brands. Thus, this finding suggests that the same mental processes as aesthetic experiences may be partly needed to develop love relationships between brands and consumers at each stage (Figure 5). This suggests aesthetic experiences may be indispensable for meaningfully developing brand-love relationships with consumers. However, our results showed that the distinctive mental processes related to the shared brain regions might function depending on each brand love developmental stage. Concretely, our results suggest that although both the functional and emotional aspects of aesthetic experiences can play a pivotal role in developing brand-love relationships during the early term, symbolic elements derived from aesthetic experiences and linking to brand-related self-knowledge build stable relationships. Marketers may need to revisit the intrinsic values of aesthetic experiences, which not only decorate shallow appearances but also lead to the bolstering of emotional bonds between brands and consumers if they consider the factors of aesthetic experience as less critical and placed at the second tier for marketing strategy.

## Data Availability

The raw data supporting the conclusions of this article will be made available by the author, without undue reservation.

## Footnotes

1
https://pubmed.ncbi.nlm.nih.gov

2
https://neuroquery.org

3
https://www.brainmap.org

4
https://mangoviewer.com

5
https://neurovault.org
