# Supplementary material for: Aesthetic evaluation underpinning brand love relationship development: an activation likelihood estimation meta-analysis and multivariate analysis
Source: Front Neurosci. 2025 Jan 3;18:1443578. doi: 10.3389/fnins.2024.1443578 (PMC11739102; doi:10.3389/fnins.2024.1443578)

Supplementary Material

Aesthetic Evaluation Underpinning Brand Love Relationship Development: An Activation Likelihood Estimation Meta-Analysis and Multivariate Analysis

**Table S1**. Overview of the studies included in the present meta-analyses.

| **First author** | **Pub Year** | **Stimulus** | **Task** | **N** |
| --- | --- | --- | --- | --- |
| Avram M | 2013 | Poem/Moral statement | Rating | 16 |
| Bohrn IC | 2013 | Sentences(proverbs) | Rating | 26 |
| Calvo-Merino B | 2008 | Movement(ballet,capoeira) | Rating | 6 |
| Carey M | 2019 | Females body | Rating | 30 |
| Chatterjee A | 2009 | Artificial face | Rating | 13 |
| Coburn A | 2020 | Achrchitectues | Passive viewing | 18 |
| Cupchik GC | 2009 | Representational paintings | Viewing orientation | 16 |
| Di Dio C | 2007 | Sculpture(classical,renaissance) | Rating | 14 |
| Faivre N | 2012 | Face(picture/movie) | Rating | 18 |
| Flexas A | 2014 | Painting(abstract,representational)/Photograph | Rating | 24 |
| Iaria G | 2008 | Face/Object | Rating | 11 |
| Ishizu T | 2011 | Paintings of portraits, landscapes/Music | Rating | 21 |
| Ishizu T | 2013 | Paintings of landscapes, portraits | Rating | 21 |
| Ishizu T | 2014 | Natural scenes(National Geographic Magazine) | Rating | 21 |
| Jacobs RH | 2012 | Visual textures | Rating | 18 |
| Jacobsen T | 2006 | Black and white patterns | Rating | 15 |
| Kawabata H | 2004 | Painting | Rating | 10 |
| Kedia G | 2014 | Full-length photographs of women and dog | Rating | 25 |
| Kirk U | 2009 | Building/Face | Rating | 22 |
| Kirk U | 2008 | Photograph(normal, abnormal) | Rating | 15 |
| Kirsch LP | 2018 | Dance movements | Rating | 48 |
| Lacey S | 2011 | Art images | Rating | 8 |
| Lebreton M | 2009 | Face/House/Painting | Rating | 20 |
| Lim SL | 2013 | Korean words | Rating | 36 |
| Martín-Loeches M | 2014 | Face/Body | Rating | 20 |
| Mizokami Y | 2014 | Painting/Photo | Rating | 39 |
| O'Doherty J | 2003 | Face | Rating | 25 |
| Pegors TK | 2015 | Face/Place | Rating | 28 |
| Racey C | 2019 | Chromatic stimuli | Passive viewing/Rating | 21 |
| Rasche SE | 2023 | Abstract painting | Rating | 18 |
| Thakral PP | 2012 | Gogh painting | Rating | 18 |
| Tsukiura T | 2011 | Face | Rating | 20 |
| Tsukiura T | 2011 | Face | Rating | 20 |
| Van der Laan LN | 2012 | Color images of food package | Rating | 20 |
| Vartanian O | 2004 | Painting(representational, abstract) | Rating | 12 |
| Vartanian O | 2013 | Photographs of architectural spaces | Rating | 18 |
| Vedder A | 2015 | Imaged environment | Imaginary task | 16 |
| Vessel EA | 2019 | Art/Architectures/Natural landscape | Rating | 18 |
| Wang T | 2015 | Drawing(face, scene) | Rating | 28/22 |
| Yang T | 2022 | Face | Rating | 19 |
| Yeh YC | 2015 | Everyday designed products | Rating | 30 |
| Zeki S | 2012 | Moving white dots | Rating | 19 |
| Zeki S | 2014 | Mathematical formulae | Rating | 15 |
| Zeng Y | 2022 | Face | Passive viewing | 17 |
| Zhang W | 2016 | Pictographs/Object image | Rating | 16 |
| Zhang W | 2017 | Pictograph/Abstract oracle bone script/grey squares | Rating | 19 |
| Zhao X | 2020 | Landscapes(dynamic, static)/Grey squares | Rating | 21 |

Abbreviations: Pub year, Publication year.

References

1. Avram, M. et al. Neurofunctional correlates of esthetic and moral judgments. Neuroscience letters 534, 128–132 (2013).

2. Bohrn, I. C., Altmann, U., Lubrich, O., Menninghaus, W. & Jacobs, A. M. When we like what we know–a parametric fMRI analysis of beauty and familiarity. Brain and Language 124, 1–8 (2013).

3. Calvo-Merino, B., Jola, C., Glaser, D. E. & Haggard, P. Towards a sensorimotor aesthetics of performing art. Consciousness and cognition 17, 911–922 (2008).

4. Carey, M., Knight, R. & Preston, C. Distinct neural response to visual perspective and body size in the extrastriate body area. Behavioural Brain Research 372, 112063 (2019).

5. Chatterjee, A., Thomas, A., Smith, S. E. & Aguirre, G. K. The neural response to facial attractiveness. Neuropsychology 23, 135 (2009).

6. Coburn, A. et al. Psychological and neural responses to architectural interiors. Cortex 126, 217–241 (2020).

7. Cupchik, G. C., Vartanian, O., Crawley, A. & Mikulis, D. J. Viewing artworks: contributions of cognitive control and perceptual facilitation to aesthetic experience. Brain and cognition 70, 84–91 (2009).

8. Di Dio, C., Macaluso, E. & Rizzolatti, G. The golden beauty: brain response to classical and renaissance sculptures. PloS one 2, e1201 (2007).

9. Faivre, N., Charron, S., Roux, P., Lehéricy, S. & Kouider, S. Nonconscious emotional processing involves distinct neural pathways for pictures and videos. Neuropsychologia 50, 3736–3744 (2012).

10. Flexas, A., Rosselló, J., de Miguel, P., Nadal, M. & Munar, E. Cognitive control and unusual decisions about beauty: an fMRI study. Frontiers in human neuroscience 8, 520 (2014).

11. Iaria, G., Fox, C. J., Waite, C. T., Aharon, I. & Barton, J. J. The contribution of the fusiform gyrus and superior temporal sulcus in processing facial attractiveness: neuropsychological and neuroimaging evidence. Neuroscience 155, 409–422 (2008).

12. Ishizu, T. & Zeki, S. Toward a brain-based theory of beauty. PloS one 6, e21852 (2011).

13. Ishizu, T. & Zeki, S. The brain’s specialized systems for aesthetic and perceptual judgment. European Journal of Neuroscience 37, 1413–1420 (2013).

14. Ishizu, T. & Zeki, S. A neurobiological enquiry into the origins of our experience of the sublime and beautiful. Frontiers in human neuroscience 8, 891 (2014).

15. Jacobs, R. H., Renken, R. & Cornelissen, F. W. Neural correlates of visual aesthetics–beauty as the coalescence of stimulus and internal state. PLoS One 7, e31248 (2012).

16. Jacobsen, T., Schubotz, R. I., Höfel, L. & Cramon, D. Y. v. Brain correlates of aesthetic judgment of beauty. Neuroimage 29, 276–285 (2006).

17. Kawabata, H. & Zeki, S. Neural correlates of beauty. Journal of neurophysiology 91, 1699–1705 (2004).

18. Kedia, G., Mussweiler, T., Mullins, P. & Linden, D. E. The neural correlates of beauty comparison. Social cognitive and affective neuroscience 9, 681–688 (2014).

19. Kirk, U., Skov, M., Christensen, M. S. & Nygaard, N. Brain correlates of aesthetic expertise: a parametric fMRI study. Brain and cognition 69, 306–315 (2009).

20. Kirk, U. The neural basis of object-context relationships on aesthetic judgment. PloS one 3, e3754 (2008).

21. Kirsch, L. P. & Cross, E. S. The influence of sensorimotor experience on the aesthetic evaluation of dance across the life span. Progress in brain research 237, 291–316 (2018).

22. Lacey, S. et al. Art for reward’s sake: Visual art recruits the ventral striatum. Neuroimage 55, 420–433 (2011).

23. Lebreton, M., Jorge, S., Michel, V., Thirion, B. & Pessiglione, M. An automatic valuation system in the human brain: evidence from functional neuroimaging. Neuron 64, 431–439 (2009).

24. Lim, S.-L., O’Doherty, J. P. & Rangel, A. Stimulus value signals in ventromedial PFC reflect the integration of attribute value signals computed in fusiform gyrus and posterior superior temporal gyrus. Journal of Neuroscience 33, 8729–8741 (2013).

25. Martín-Loeches, M., Hernández-Tamames, J. A., Martín, A. & Urrutia, M. Beauty and ugliness in the bodies and faces of others: an fMRI study of person esthetic judgement. Neuroscience 277, 486–497 (2014).

26. Mizokami, Y. et al. Difference in brain activations during appreciating paintings and photographic analogs. Frontiers in human neuroscience 8, 478 (2014).

27. O’Doherty, J. et al. Beauty in a smile: the role of medial orbitofrontal cortex in facial attractiveness. Neuropsychologia 41, 147–155 (2003).

28. Pegors, T. K., Kable, J. W., Chatterjee, A. & Epstein, R. A. Common and unique representations in pFC for face and place attractiveness. Journal of cognitive neuroscience 27, 959–973 (2015).

29. Racey, C., Franklin, A. & Bird, C. M. The processing of color preference in the brain. Neuroimage 191, 529–536 (2019).

30. Rasche, S. E., Beyh, A., Paolini, M. & Zeki, S. The neural determinants of abstract beauty. European Journal of Neuroscience (2023) doi:https://doi.org/10.1111/ejn.15912.

31. Thakral, P. P., Moo, L. R. & Slotnick, S. D. A neural mechanism for aesthetic experience. Neuroreport 23, 310–313 (2012).

32. Tsukiura, T. & Cabeza, R. Shared brain activity for aesthetic and moral judgments: implications for the Beauty-is-Good stereotype. Social cognitive and affective neuroscience 6, 138–148 (2011).

33. Tsukiura, T. & Cabeza, R. Remembering beauty: Roles of orbitofrontal and hippocampal regions in successful memory encoding of attractive faces. Neuroimage 54, 653–660 (2011).

34. Van der Laan, L. N., De Ridder, D. T., Viergever, M. A. & Smeets, P. A. Appearance matters: neural correlates of food choice and packaging aesthetics. PloS one 7, e41738 (2012).

35. Vartanian, O. & Goel, V. Neuroanatomical correlates of aesthetic preference for paintings. Neuroreport 15, 893–897 (2004).

36. Vartanian, O. et al. Impact of contour on aesthetic judgments and approach-avoidance decisions in architecture. Proceedings of the National Academy of Sciences 110, 10446–10453 (2013).

37. Vedder, A. et al. Neurofunctional correlates of environmental cognition: an fMRI study with images from episodic memory. PloS one 10, e0122470 (2015).

38. Vessel, E. A., Isik, A. I., Belfi, A. M., Stahl, J. L. & Starr, G. G. The default-mode network represents aesthetic appeal that generalizes across visual domains. Proceedings of the National Academy of Sciences 116, 19155–19164 (2019).

39. Wang, T. et al. Is moral beauty different from facial beauty? Evidence from an fMRI study. Social cognitive and affective neuroscience 10, 814–823 (2015).

40. Yeh, Y., Lin, C.-W., Hsu, W.-C., Kuo, W.-J. & Chan, Y.-C. Associated and dissociated neural substrates of aesthetic judgment and aesthetic emotion during the appreciation of everyday designed products. Neuropsychologia 73, 151–160 (2015).

41. Yang, T., Formuli, A., Paolini, M. & Zeki, S. The neural determinants of beauty. European Journal of Neuroscience 55, 91–106 (2022).

42. Zeki, S. & Stutters, J. A brain-derived metric for preferred kinetic stimuli. Open biology 2, 120001 (2012).

43. Zeki, S., Romaya, J. P., Benincasa, D. M. & Atiyah, M. F. The experience of mathematical beauty and its neural correlates. Frontiers in human neuroscience 8, 68 (2014).

44. Zeng, Y. et al. Symmetric in the striate but asymmetric in the extrastriate cortex when processing three-quarter faces: Neural underpinnings for aesthetic appreciations. PsyCh Journal 11, 720–728 (2022).

45. Zhang, W., Lai, S., He, X., Zhao, X. & Lai, S. Neural correlates for aesthetic appraisal of pictograph and its referent: An fMRI study. Behavioural Brain Research 305, 229–238 (2016).

46. Zhang, W. et al. Neural substrates of embodied natural beauty and social endowed beauty: an fMRI study. Scientific Reports 7, 1–12 (2017).

47. Zhao, X. et al. The neural mechanism of aesthetic judgments of dynamic landscapes: an fMRI study. Scientific Reports 10, 1–11 (2020).

**Table S2**. Peak coordinates in brain regions related to aesthetic experiences.

| Cluster | Side | Brain region | BA | Coordinates | | | ALE values | Cluster Size (mm3) |
| --- | --- | --- | --- | --- | --- | --- | --- | --- |
|  |  |  |  | x | y | z |  |  |
| 1 | R | Inferior Occipital Gyrus | BA17 | 24 | -98 | -6 | 0.0817 | 11352 |
|  | R | Middle Occipital Gyrus | BA18 | 30 | -90 | -6 | 0.0755 |  |
|  | R | Fusiform Gyrus | BA19 | 44 | -72 | -10 | 0.0398 |  |
|  | R | Fusiform Gyrus | BA37 | 40 | -52 | -18 | 0.0369 |  |
|  | R | Cerebellum(Declive) | --- | 38 | -58 | -12 | 0.0355 |  |
|  | R | Lingual Gyrus | BA18 | 38 | -78 | -4 | 0.0319 |  |
|  | R | Cerebellum(Culmen) | --- | 30 | -48 | -14 | 0.0264 |  |
|  | R | Cerebellum(Declive) | --- | 30 | -68 | -12 | 0.0241 |  |
| 2 | L | Anterior Cingulate (VMPFC) | BA24 | -4 | 40 | -8 | 0.0492 | 5624 |
|  | L | Medial Frontal Gyrus (MPFC) | BA10 | -4 | 54 | -2 | 0.0475 |  |
|  | R | Anterior Cingulate (VMPFC) | BA24 | 6 | 34 | -10 | 0.0310 |  |
| 3 | L | Medial Frontal Gyrus (DMPFC) | BA6 | -4 | 22 | 44 | 0.0887 | 4832 |
| 4 | L | Inferior Occipital Gyrus | BA18 | -32 | -90 | -8 | 0.0911 | 4312 |
|  | L | Fusiform Gyrus | BA19 | -40 | -80 | -10 | 0.0303 |  |
|  | L | Fusiform Gyrus | BA19 | -40 | -72 | -14 | 0.0231 |  |
| 5 | L | Cerebellum(Culmen) | --- | -38 | -50 | -22 | 0.0412 | 2616 |
|  | L | Parahippocampal Gyrus | BA37 | -28 | -46 | -10 | 0.0389 |  |
| 6 | L | Inferior Frontal Gyrus (DLPFC) | BA9 | -44 | 8 | 28 | 0.0661 | 2496 |
| 7 | R | Inferior Frontal Gyrus (DLPFC) | BA9 | 46 | 12 | 26 | 0.0835 | 2392 |
| 8 | R | Lingual Gyrus | BA17 | 12 | -92 | 2 | 0.0318 | 1680 |
|  | R | Lingual Gyrus | BA17 | 14 | -92 | 10 | 0.0306 |  |
| 9 | R | Claustrum | --- | 34 | 22 | 0 | 0.0374 | 1248 |

**Table 3 (a).** Decoded results by the Neurosynth cognitive decoding.

| **Rank** | **Term** | **Mental process constructs** | **corr** |
| --- | --- | --- | --- |
| **Overlapping brain regions: Early stage of brand love & Aesthetic experiences** | | | |
| 1 | value | RD | 0.237 |
| 2 | money | RD | 0.217 |
| 3 | midline | Self | 0.19 |
| 4 | referential | Self | 0.155 |
| 5 | default | DMN | 0.154 |
| 6 | default mode | DMN | 0.149 |
| 7 | self referential | Self | 0.145 |
| 8 | smoking | RD | 0.144 |
| 9 | mood | EM | 0.142 |
| 10 | subjective | Self | 0.127 |
| 11 | engagement | UD | 0.126 |
| 12 | valence | EM | 0.121 |
| 13 | personal | UD | 0.121 |
| 14 | social interactions | SC | 0.114 |
| 15 | rewards | RD | 0.113 |
| **Overlapping brain regions: Migration stage of brand love & Aesthetic experiences** | | | |
| 1 | value | RD | 0.246 |
| 2 | money | RD | 0.207 |
| 3 | midline | Self | 0.198 |
| 4 | referential | Self | 0.154 |
| 5 | default | DMN | 0.149 |
| 6 | default mode | DMN | 0.144 |
| 7 | mood | EM | 0.14 |
| 8 | self referential | Self | 0.138 |
| 9 | smoking | RD | 0.137 |
| 10 | personal | UD | 0.126 |
| 11 | choose | RD | 0.122 |
| 12 | subjective | Self | 0.121 |
| 13 | engagement | UD | 0.12 |
| 14 | social interactions | SC | 0.116 |
| 15 | valence | EM | 0.114 |
| **Overlapping brain regions: Stable stage of brand love & Aesthetic experiences** | | | |
| 1 | similarity | SM | 0.361 |
| 2 | engagement | UD | 0.243 |
| 3 | letter | SM | 0.232 |
| 4 | color | UD | 0.17 |
| 5 | performance | CW | 0.169 |
| 6 | judgment | CW/SL | 0.158 |
| 7 | forms | UD | 0.155 |
| 8 | demands | CW | 0.142 |
| 8 | interference | CW | 0.142 |
| 9 | stroop | CW | 0.139 |
| 10 | control task | CW | 0.138 |
| 11 | maintenance | CW | 0.129 |
| 12 | difficulty | CW | 0.123 |
| 13 | verbal | SL | 0.113 |
| 14 | phonological | SL | 0.113 |
| 15 | switch | CW | 0.109 |

Abbreviations: corr, correlation coefficient; CW, cognitive control and working memory; DMN, default mode network; EM, emotion; RD, reward-based decision-making; SC, social cognition; Self, self-referential; SL, semantic and language; UD, undefined.

**Table 3 (b).** Decoded results by the NeuroQuery decoding model.

| **Rank** | **Term** | **Mental process constructs** | **Similarity score** |
| --- | --- | --- | --- |
| **Overlapping brain regions: Early stage of brand love & Aesthetic experiences** | | | |
| 1 | choice | RD | 0.94 |
| 2 | conflict | CW | 0.82 |
| 3 | cognitive control | CW | 0.8 |
| 4 | borne | RD | 0.69 |
| 5 | evaluation | CW/EM/RD | 0.67 |
| 6 | mother | UD | 0.64 |
| 7 | setting | UD | 0.63 |
| 8 | social cognitive | SC | 0.62 |
| 9 | trait | UD | 0.61 |
| 10 | economic | RD | 0.6 |
| 10 | emotional responses | EM | 0.6 |
| 11 | rule | UD | 0.59 |
| 12 | sorting | CW | 0.58 |
| 12 | game | RD | 0.58 |
| 12 | stroop | CW | 0.58 |
| 13 | inappropriate | CW/RD/SL | 0.57 |
| 13 | strategy | UD | 0.57 |
| 14 | wisconsin | CW | 0.55 |
| 15 | negative emotion | EM | 0.54 |
| 15 | norm | SL | 0.54 |
| **Overlapping brain regions: Migration stage of brand love & Aesthetic experiences** | | | |
| 1 | choice | RD | 0.75 |
| 2 | borne | RD | 0.65 |
| 3 | trait | UD | 0.6 |
| 4 | social cognitive | SC | 0.55 |
| 4 | default | DMN | 0.55 |
| 5 | emotional responses | EM | 0.52 |
| 5 | evaluation | CW/EM/RD | 0.52 |
| 5 | mother | UD | 0.52 |
| 6 | mood | EM | 0.51 |
| 7 | positive negative | EM | 0.49 |
| 7 | self referential | Self | 0.49 |
| 8 | impact | DMN/RD | 0.48 |
| 8 | referential | Self | 0.48 |
| 8 | positive | EM | 0.48 |
| 9 | price | RD | 0.45 |
| 9 | network | DMN | 0.45 |
| 9 | gift | RD | 0.45 |
| 10 | midline | Self | 0.44 |
| 10 | health behavior | RD/Self | 0.44 |
| 11 | sedentary | RD/Self | 0.42 |
| 11 | default network | DMN | 0.42 |
| 11 | abuse | RD | 0.42 |
| 12 | inappropriate | CW/RD/SL | 0.41 |
| 12 | disinhibition | CW/RD | 0.41 |
| 13 | products | RD | 0.4 |
| 13 | willingness | RD | 0.4 |
| 13 | altruistic | SC | 0.4 |
| 14 | thought | DMN | 0.39 |
| 14 | public | UD | 0.39 |
| 14 | currently | DMN/RD | 0.39 |
| 14 | failure | DMN/RD | 0.39 |
| 15 | network dmn | DMN | 0.38 |
| 15 | induction | DMN | 0.38 |
| 15 | dmn | DMN | 0.38 |
| **Overlapping brain regions: Stable stage of brand love & Aesthetic experiences** | | | |
| 1 | left | UD | 1 |
| 2 | task | CW | 0.98 |
| 3 | syntactic | SL | 0.81 |
| 4 | demand | CW | 0.79 |
| 4 | switching | CW | 0.79 |
| 5 | reaction time | CW | 0.78 |
| 5 | fluency | CW/SL | 0.78 |
| 6 | distractor | CW | 0.77 |
| 7 | verbal fluency | CW/SL | 0.75 |
| 7 | semantic | SL | 0.75 |
| 7 | sets | UD | 0.75 |
| 8 | word | SL | 0.74 |
| 8 | letter | CW | 0.74 |
| 8 | reaction | UD | 0.74 |
| 8 | german | SL | 0.74 |
| 8 | resource | CW | 0.74 |
| 9 | dual | CW | 0.73 |
| 10 | meaning | SL | 0.72 |
| 11 | task demands | CW | 0.71 |
| 11 | generation | CW/SL | 0.71 |
| 12 | difficult | CW | 0.7 |
| 12 | judgment task | CW/SL | 0.7 |
| 13 | vocabulary | CW/SL | 0.69 |
| 14 | semantic processing | SL | 0.68 |
| 14 | accuracy | CW | 0.68 |
| 14 | task difficulty | CW | 0.68 |
| 15 | task switching | CW | 0.67 |
| 15 | syntax | SL | 0.67 |
| 15 | phonological | SL | 0.67 |
| 15 | list | SL | 0.67 |

Abbreviations: CW, cognitive control and working memory; DMN, default mode network; EM, emotion; RD, reward-based decision-making; SC, social cognition; Self, self-referential; SL, semantic and language; UD, undefined.

**Table 3 (c).** Decoded results by the Behavioral Analysis Plugin.

| **Category** | **Domain** | **Z-score** |
| --- | --- | --- |
| **Overlapping brain regions: Early stage of brand love & Aesthetic experiences** | | |
| Attention | Cognition | 14.2160 |
| Reasoning | Cognition | 11.0608 |
| Positive (Reward/Gain) | Emotion | 10.2951 |
| Language (Semantics) | Cognition | 10.0657 |
| Memory (Working) | Cognition | 8.9042 |
| Memory (Expliicit) | Cognition | 8.8482 |
| Language (Speech) | Cognition | 8.3024 |
| Vision (Shape) | Perception | 7.9352 |
| Inhibition | Action | 7.8494 |
| Spatial | Cognition | 5.9971 |
| Negative (Fear) | Emotion | 5.7608 |
| Vision (Unspecified) | Perception | 5.7298 |
| Somesthesis (Pain) | Perception | 5.7203 |
| Language (Phonology) | Cognition | 5.6913 |
| Social Cognition | Cognition | 5.2519 |
| Language (Orthography) | Cognition | 5.0887 |
| Sexuality | Interoception | 4.9717 |
| Music | Cognition | 4.8833 |
| Negative (Unspecified) | Emotion | 4.8477 |
| Audition | Perception | 4.6821 |
| Somesthesis (Unspecified) | Perception | 4.4289 |
| Observation | Action | 3.9739 |
| Positive (Unspecified) | Emotion | 3.8943 |
| Execution (Unspecified) | Action | 3.8931 |
| Negative (Disgust) | Emotion | 3.7348 |
| Gustation | Perception | 3.5216 |
| Execution (Speech) | Action | 3.4790 |
| **Overlapping brain regions: Migration stage of brand love & Aesthetic experiences** | | |
| Positive (Reward/Gain) | Emotion | 6.4091 |
| Reasoning | Cognition | 6.3380 |
| Attention | Cognition | 4.9880 |
| Negative (Fear) | Emotion | 4.0117 |
| Memory (Expliicit) | Cognition | 3.7151 |
| Social Cognition | Cognition | 3.3839 |
| Sexuality | Interoception | 3.3398 |
| **Overlapping brain regions: Stable stage of brand love & Aesthetic experiences** | | |
| Memory (Working) | Cognition | 4.8676 |
| Language (Semantics) | Cognition | 4.4059 |
| Reasoning | Cognition | 4.3944 |
| Language (Phonology) | Cognition | 4.2913 |
| Language (Speech) | Cognition | 4.0113 |
| Attention | Cognition | 3.8788 |
| Language (Orthography) | Cognition | 3.0807 |

**Figure S1.** Activated brain regions of Aesthetic experiences.


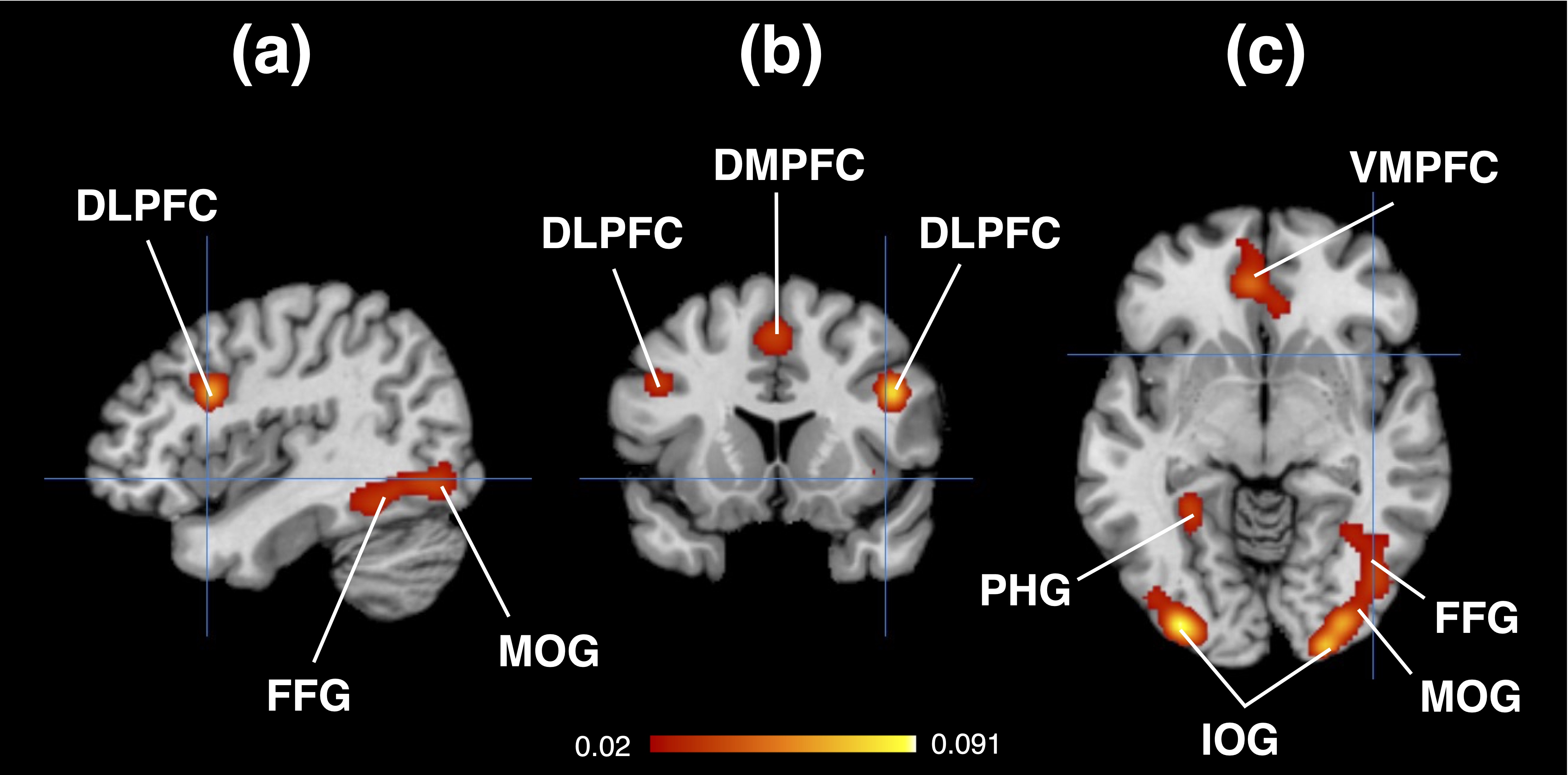


Crosshairs = (42 14 -8). **(a)** Sagittal view. **(b)** Coronal view. **(c)** Axial view. Abbreviations: DLPFC, dorsal lateral prefrontal cortex; DMPFC, dorsal medial prefrontal cortex; FFG, fusiform gyrus; IOG, inferior occipital gyrus; MOG, middle occipital gyrus; PHG, parahippocampal gyrus; VMPFC, ventral medial prefrontal cortex.

**Figure S2.** Meta-analysis modules in the Neurosynth. **(a)** Topic-based meta-analysis module. **(b)** Term-based meta-analysis module.


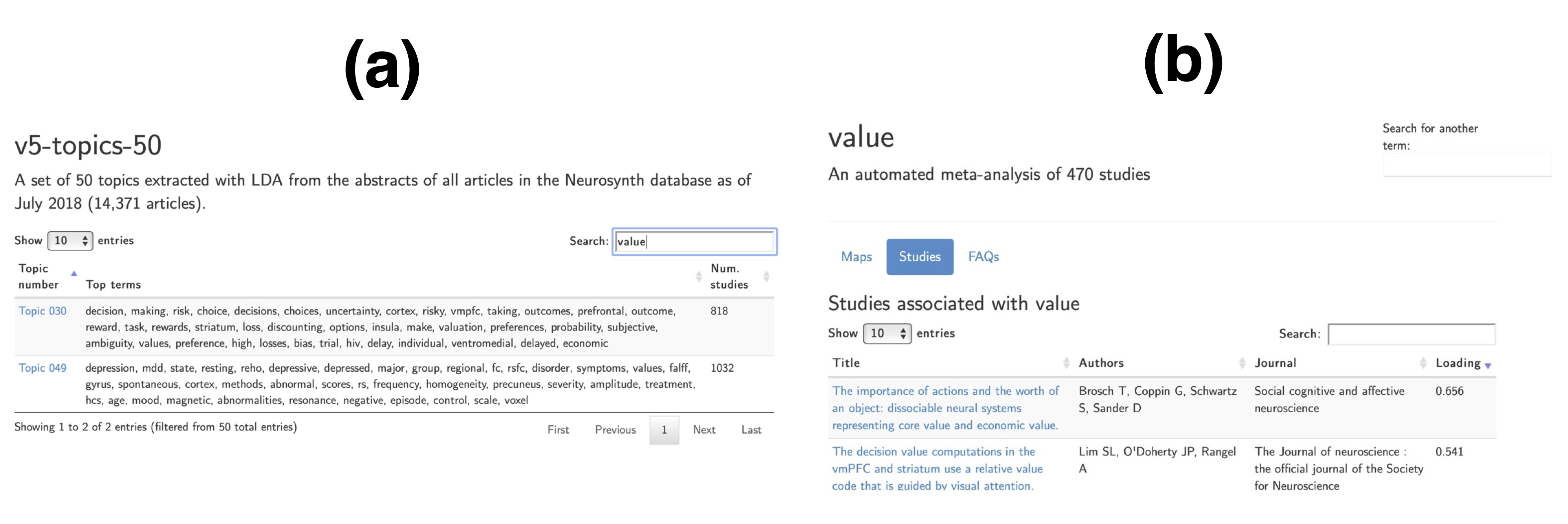


**Figure S3.** Translating decoded terms using the NeuroQuery. **(a)** Input a decoded term. **(b)** “In expansion” section. **(c)** “Publications related to the query” section. 30 related studies are listed.


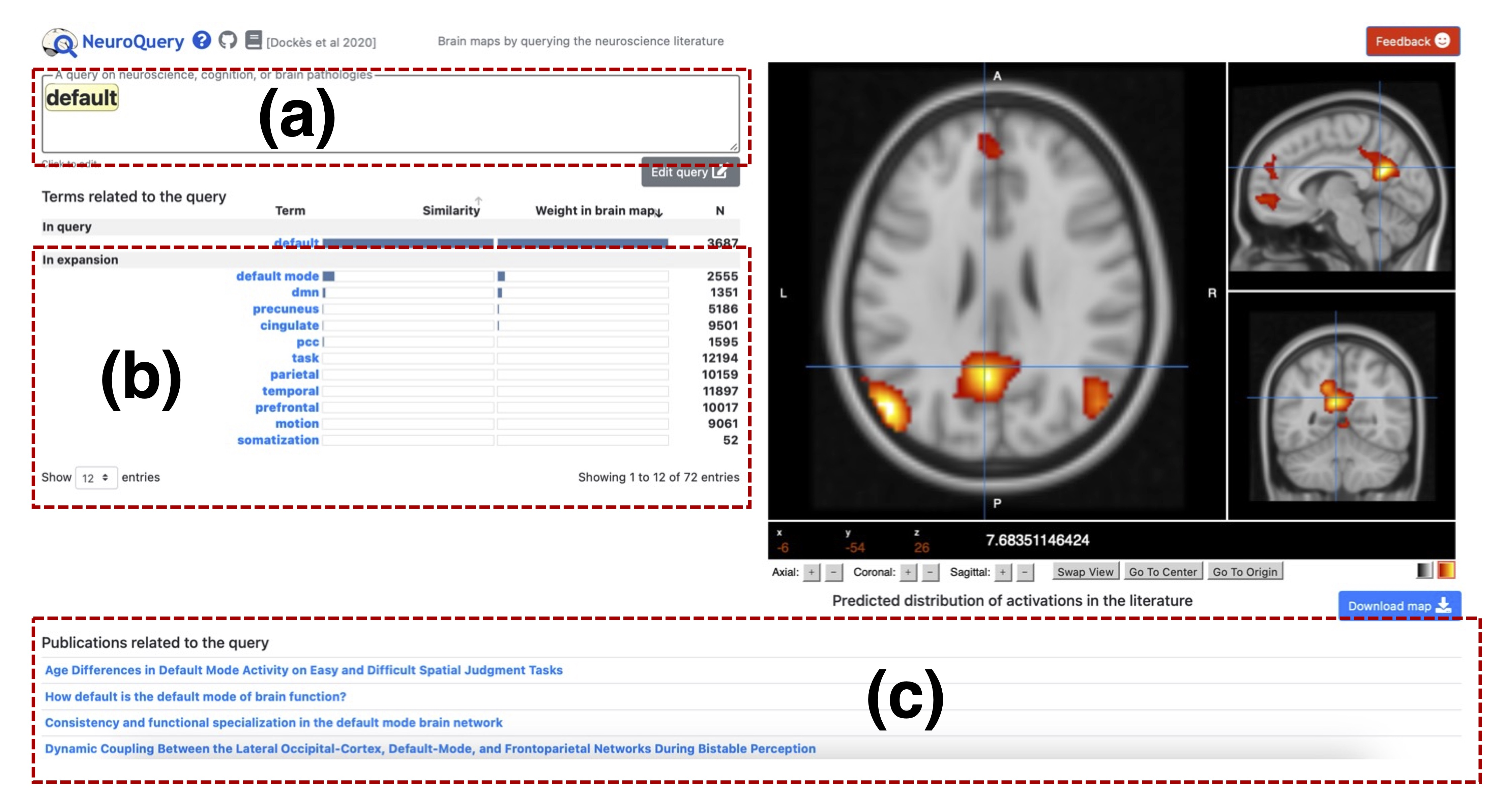

Supplement: Supplementary file 1 [file Data_Sheet_1.docx]
